# MicroRNA Modulation Induced by AICA Ribonucleotide in J1 Mouse ES Cells

**DOI:** 10.1371/journal.pone.0103724

**Published:** 2014-07-31

**Authors:** Xiaoyan Shi, YongyanWu, Zhiying Ai, Juan Du, Lixia Cao, Zekun Guo, Yong Zhang

**Affiliations:** 1 College of Life Sciences, Northwest A&F University, Yangling, Shaanxi, China; 2 College of Veterinary Medicine, Northwest A&F University, Yangling, Shaanxi, China; 3 Key Laboratory of Animal Biotechnology, Ministry of Agriculture, Northwest A&F University, Yangling, Shaanxi, China; Nazarbayev University, Kazakhstan

## Abstract

ES cells can propagate indefinitely, maintain self-renewal, and differentiate into almost any cell type of the body. These properties make them valuable in the research of embryonic development, regenerative medicine, and organ transplantation. MicroRNAs (miRNAs) are considered to have essential functions in the maintenance and differentiation of embryonic stem cells (ES cells). It was reported that, strong external stimuli, such as a transient low-pH and hypoxia stress, were conducive to the formation of induced pluripotent stem cells (iPS cells). AICA ribonucleotide (AICAR) is an AMP-activated protein kinase activator, which can let cells in the state of energy stress. We have demonstrated that AICAR can maintain the pluripotency of J1 mouse ES cells through modulating protein expression in our previous research, but its effects on ES cell miRNA expression remain unknown. In this study, we conducted small RNA high-throughput sequencing to investigate AICAR influence on J1 mouse ES cells by comparing the miRNA expression patterns of the AICAR-treated cells and those without treatment. The result showed that AICAR can significantly modulate the expression of multiple miRNAs, including those have crucial functions in ES cell development. Some differentially expressed miRNAs were selected and confirmed by real-time PCR. For the differently expressed miRNAs identified, further study was conducted regarding the pluripotency and differentiation associated miRNAs with their targets. Moreover, miR-134 was significantly down-regulated after AICAR treatment, and this was suggested to be directly associated with the up-regulated pluripotency markers, Nanog and Sox2. Lastly, Myc was significantly down-regulated after AICAR treatment; therefore, we predicted miRNAs that may target Myc and identified that AICAR induced up-regulation of miR-34a, 34b, and 34c can repress Myc expression in J1 mouse ES cells. Taken together, our study provide a new mechanism for AICAR in ES cells pluripotency maintenance and give insight for its usage in iPS cells generation.

## Introduction

Embryonic stem cells (ES cells) are blastocyst inner cell mass-derived cells that are pluripotent and capable of self-renewal and immortalization [1]. Therefore, ES cells are usually considered as a better system to study embryonic development, regenerative medicine, and tissue replacement. In ES cells, the balance between pluripotency and differentiation should be strictly controlled. The core transcription network composed of Nanog, Oct4, Klf4, and Sox2 is known to be essential for ES cell self-renewal and pluripotency maintenance [2]. Other signal pathways, including Wnt, TGF-β, LIF/JAK-STAT, and MEK/ERK signal pathway, also have crucial functions in ES cells [3]–[6]. However, this homeostasis is influenced by the regulation of epigenetic modifications as well. As an important mechanism of epigenetic regulation, microRNAs (miRNAs) play crucial roles in the regulation of ES cell fate decision [7].

MiRNAs contain a class of non-coding RNAs that are widely expressed in both plants and animals [8]. They are usually transcribed by RNA polymerase II (Pol II) to generate a stem loop containing primary miRNA (pri-miRNA) and processed by DGCR8/Pasha complex to produce ∼70 nt hairpin precursor miRNA (pre-miRNA). Thereafter, the pre-miRNAs are transported into the cytoplasm, where they are cleaved and processed to become mature miRNAs [9]. MiRNAs generally function in cytoplasm by near-perfect or perfect base pair with their target mRNAs in animals or plants, respectively, to promote translation repression or induce mRNA degradation or cleavage. Notably, a single miRNA can regulate the expression of multiple target mRNAs; simultaneously, a single mRNA can be regulated by several miRNAs. Therefore, the miRNA pathway is a novel and complicated regulation network that influences a large amount of biological processes [10]. Over the past decade, substantial amount of research have been conducted on expounding miRNA expression and function in the differentiation and maintenance of ES cells. As a result, a large number of ES cell-specific miRNAs, such as the members of miR-290 cluster, have been identified as important for ES cell stemness [11].

Small molecules, including Pluripotin/SC1, CHIR9921, SB431542, and BIO, have been demonstrated to manipulate the fate of ES cells by promoting pluripotency or inhibiting differentiation through modulating diverse signal transduction pathways [12]–[15]. Moreover, Hou *et al* have first reported that iPS cells can be generated from mouse somatic cells using a combination of seven small molecule compounds [16]. Recently, this suggestion was also demonstrated by a research team from Korea and they produced iPS-like cells by the combination of other small molecules to compensate for reprogramming factors [17]. Obokata has demonstrated that strong external stimulus, such as transient low-pH stress or other forms of stress, can facilitate the generation of iPS cells from mammalian somatic cells [18]. This reprogramming phenomenon was named as stimulus-triggered acquisition of pluripotency (STAP), and it provided a convenient and efficient pathway for iPS cells generation. Ou *et al* also reported that, in the condition of hypoxia stress, SIRT1 can modulate the autophagy of ES cells as well as their mitochondria function [19]. These studies indicated that a certain degree of external stimuli or stress can influence ES cell pluripotency maintenance and intracellular metabolism.

AICAR can lead increased levels of intracellular AMP and influence cellular energy metabolism. This function was similarly to FSK, which was one of the small molecules used by Hou to generate iPS cells. Considering its function in energy stress and potential usage in iPS generation, we choose AICAR as our research target. Furthermore, we have reported previously that AICAR can elevate the expression of pluripotency-associated genes and maintain ES cell stemness [20], but its function in ES cells miRNAs modulation was unclear. In this study, we performed small RNA (sRNA) high-throughput sequencing to identify differentially expressed miRNAs of J1 ES cells cultured with or without AICAR, so as to discover the mechanism of AICAR in ES cell miRNAs modulation and stemness maintenance. These data will provide new evidence for STAP and give insight for the application of AICAR in somatic cell reprogramming.

## Materials and Methods

### Cell culture

All cell culture reagents and sterile cell wells were purchased from Gibco (Invitrogen, Carlsbad, CA, USA) and Nunclon (Roskilde, Denmark), respectively, unless otherwise indicated. J1 mouse ES cells were purchased from ATCC (Manassas, VA, USA) and maintained as previously described [16]. 293FT cell line was cultured at 37°C humidified air with 5% CO_2_ in Dulbecco’s modified Eagle medium supplemented with 10% fetal bovine serum.

### AICAR treatment

AICAR (Beyotime Institute of Biotechnology, Jiangsu, China) was dissolved in dimethyl sulfoxide (DMSO), and their final working concentration was 1 mM. Cell medium with an equal volume of DMSO was used as control.

### RNA isolation and sRNA high-throughput sequencing of J1 ES cells

Total RNA was extracted using TRIZOL reagent (Life Technologies, Carlsbad, CA, USA) by following the manufacturer’s instructions. Total RNA samples were subjected to size fractionation to obtain 18 nt to 30 nt sRNAs. Then, these sRNAs were added with 3′ and 5′ adaptor and reverse transcribed to double strand cDNA. These sequences were amplified through PCR and subjected to high-throughput sequencing. The 50 nt sequence tags from HiSeq sequencing initially underwent data cleaning, which includes the removal of low-quality tags and several kinds of contaminant reads. Finally, the clean reads were obtained and applied to the analysis. The list of significantly regulated microRNAs were filtered using the threshold of p-value≤0.05 and fold change (FC)≥2 or ≤0.5 between AICAR-treated J1 ES cells compared with those added with DMSO.

Small RNA sequencing data were deposited in the NCBI GEO database (http://www.ncbi.nlm.nih.gov/geo/query/acc.cgi?acc=GSE54586).

### RT-PCR and quantitative real-time PCR analysis

Validation of the sRNA high-throughput sequencing data was performed using quantitative real-time PCR analysis with the ABI StepOnePlus PCR system (Applied Biosystems, Foster City, CA, USA). For miRNA analysis, the total RNA (2 µg) was reverse transcribed using a miScript II RT kit (Qiagen China Shanghai Co. Ltd.) with 5 × miScript HiSpec buffer to obtain mature miRNA. Real-time PCR was performed in triplicate for each sample using miScript SYBR Green PCR kit (Qiagen China Shanghai Co. Ltd.). All reactions were performed at 95°C for 15 min to activate the HotStarTaq DNA polymerase. This process was followed by 40 cycles of 95°C for 5 s and 60°C for 30 s, which ends with a melting curve acquisition. We used the endogenous reference RNA RNU6B to normalize the amount of template added. The 2^−ΔΔCT^ method was used to analyze relative changes in miRNA expression, and a reference sample was performed in each independent experiment. The detection of gene expression by real-time PCR was performed as previously described [16]. The primer sequences used for real-time PCR are shown in Table S1.

### Vector construction

The 3′-UTRs of Myc that contain putative miRNA binding sites were amplified through PCR from J1 ES cell cDNA and cloned into multiple cloning sites (MCS) of psiCHECK-2 Vector (Promega). MiRNA expression vectors were constructed by inserting PCR-amplified pre-miRNA into MCS of pCDH-CMV-MCS-EF1-copGFP (System Biosciences, CA, USA).

### Dual-luciferase reporter assay

J1 ES cells were seeded on gelatin-coated 24-well cell plates one day before transfection. When the cells obtained 50% to 60% confluence, miRNA expression vectors and luciferase reporters that contained 3′-UTR of Myc were co-transfected by Lipofectamine 2000 (Life Technologies, Carlsbad, CA, USA) according to the manufacturer’s protocol. Then, 24 or 48 hours after transfection, the cells were lysed in 1 × passive lysis buffer. Luciferase activity was measured with the dual-luciferase reporter assay system (Promega) according to the manufacturer’s instructions on a Victor X5 multi-label plate reader (PerkinElmer, Norwalk, CT, USA).

### Alkaline phosphatase activity assay

J1 ES cells were seeded on gelatin-coated 12-well plates in the presence of 1,000 U/ml LIF and transfected with miR-134 expression vector pCDH-mir134 or its negative control. 12 h after transfection, AICAR or equal volume of DMSO were added into cell medium. After 24 h, the alkaline phosphatase activity of cells cultured in defined medium was detected with the BCIP/NBT alkaline phosphatase color development kit (Beyotime Institute of Biotechnology) following the manufacturer’s instructions.

### Western blot analysis

J1 ES cells were treated with DMSO or AICAR for 24 h on gelatin-coated 6-well plates and total cell lysates were extracted with RIPA buffer. Proteins were separated by 10% acrylamide gels and transferred to PVDF membranes (Millipore, MA, USA) for 2.5 h at 100 V. Then, the membranes were blocked in 5% non-fat milk/TBST for 2 h and incubated with the primary antibody overnight at 4°C. After that, membranes were washed three times with TBST and incubated with the secondary antibody for 2 h. Finally, membranes were washed three times for 10 min each with TBST, and immunoblots were developed by autography using SuperSignal west pico substrate (Thermo Scientific, IL, USA).

### Statistical analysis

Data were presented as the mean ± standard deviation (SD), and statistical significances were analyzed using the Student’s t-test. A value of p<0.05 was considered significant.

## Results

### Overview of sRNA high-throughput sequencing data

Small non-coding RNAs belong to a class of functional RNA that is not translated into protein. Small RNA high-throughput sequencing is strong because of its decreased nucleotide loss, small sample quantity requirement, high throughput, and high accuracy. Therefore, an increasing number of researchers choose this method as a powerful tool in small RNA function research.

**Figure 1:**
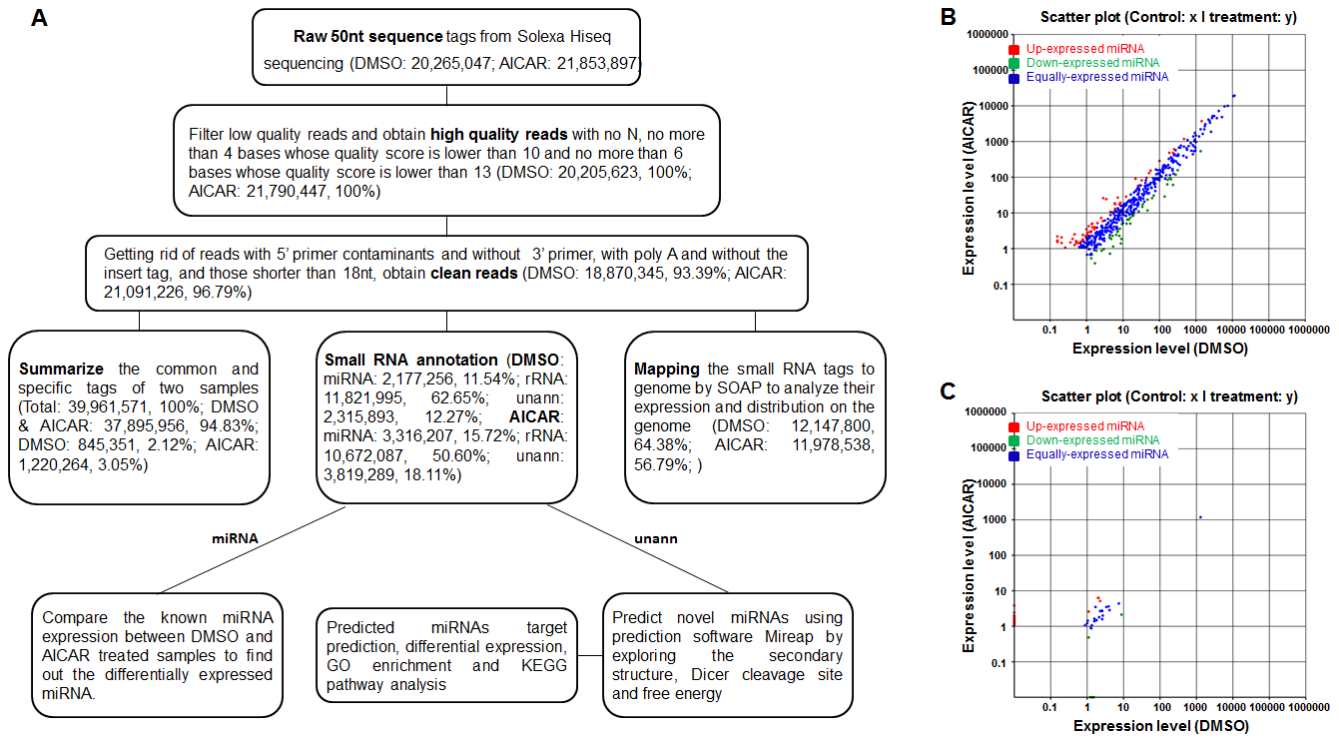
Overview of sRNA high-throughput sequencing data. (A) A flowchart that shows stepwise data analysis process of small RNA reads from Solexa Hiseq sequencing. (B) Comparison of the known miRNA expression between DMSO- and AICAR-treated samples; the scatter plot shows differentially expressed miRNAs in the two samples. (C) Comparison of the predicted novel miRNA expression between DMSO- and AICAR-treated samples; the scatter plot shows differentially expressed miRNAs in the two samples.

In this study, we treated J1 ES cells with 1 mM AICAR or equal volume of DMSO for 24 h and isolated total RNA using trizol reagent. Extracted total RNA samples were subjected to size filtration, and only those between 18 and 30 nt could pass through. The filtered sRNA was added with 3′ and 5′ adaptor, reverse transcribed, PCR amplified, and subjected to Solexa high-throughput sequencing. The stepwise data analysis process of sRNA reads from Solexa Hiseq sequencing was shown in Figure 1A and only the filtered clean reads were used to further analysis. The small RNA tags were mapped into the genome by short oligonucleotide alignment program (SOAP), which can analyze the expression and distribution of the clean reads in the genome. This finding revealed that approximately 64.38% (12,147,800) clean reads of DMSO and 56.79% (11,978,538) clean reads of AICAR can be mapped into the genome. Furthermore, these mapping reads were distributed among all 40 mouse chromosomes, as well as both the sense and anti-sense of these chromosomes.

Small RNA annotation is the most important process in our work. Through this procedure, we identified 2,177,256 (11.54% of clean reads) and 3,316,207 (15.72% of clean reads) miRNAs from DMSO- and AICAR-treated samples, respectively. Furthermore, we compared the expression of those identified miRNA between the AICAR-treated sample and the DMSO-treated sample. An overview of differentially expressed miRNAs is shown by the scatter plot in Figure 1B. Aside from those annotated sRNAs, some unannotated small RNA tags were also noted (DMSO: 2,315,893, 12.27%; AICAR: 3,819,289, 18.11%). We predict novel miRNAs using prediction software Mireap by exploring the secondary structure, as well as Dicer cleavage site and the minimum free energy of the unannotated sRNA tags that can be mapped into the genome. Finally, we predicted 149 kinds of novel miRNA in the AICAR-treated sample and 129 kinds of novel miRNA in the DMSO-treated sample. We also identified several differentially expressed novel miRNAs between the DMSO- and AICAR -treated samples, and the result is presented in Figure 1C.

Overall, we obtained a sufficient amount of data from sRNA high-throughput sequence for our subsequent analysis. We found that the addition of AICAR significantly modulated the expression of a large number of known miRNAs and predicted novel miRNAs, which is further demonstrated in the following parts of this paper.

### AICAR modulates the expression of multiple miRNAs associated with ES cell pluripotency

We have previously demonstrated that AICAR could stimulate J1 ES cell self-renewal and pluripotency by regulating protein-encoding gene expression. However, the mechanism by which AICAR modulates miRNA expression has not been elaborated. In this study, we performed high-throughput sRNA sequencing and compared differentially expressed miRNAs of J1 ES cells with and without AICAR treatment. With the addition of 1 mM AICAR, we observed that 43 miRNAs were significantly down-regulated (FC≤0.5, p≤0.01) and 59 miRNAs were significantly up-regulated (FC≥2, p≤0.01) compared with those cultured in the equal volume of DMSO (Tables S2 and S3).

**Figure 2:**
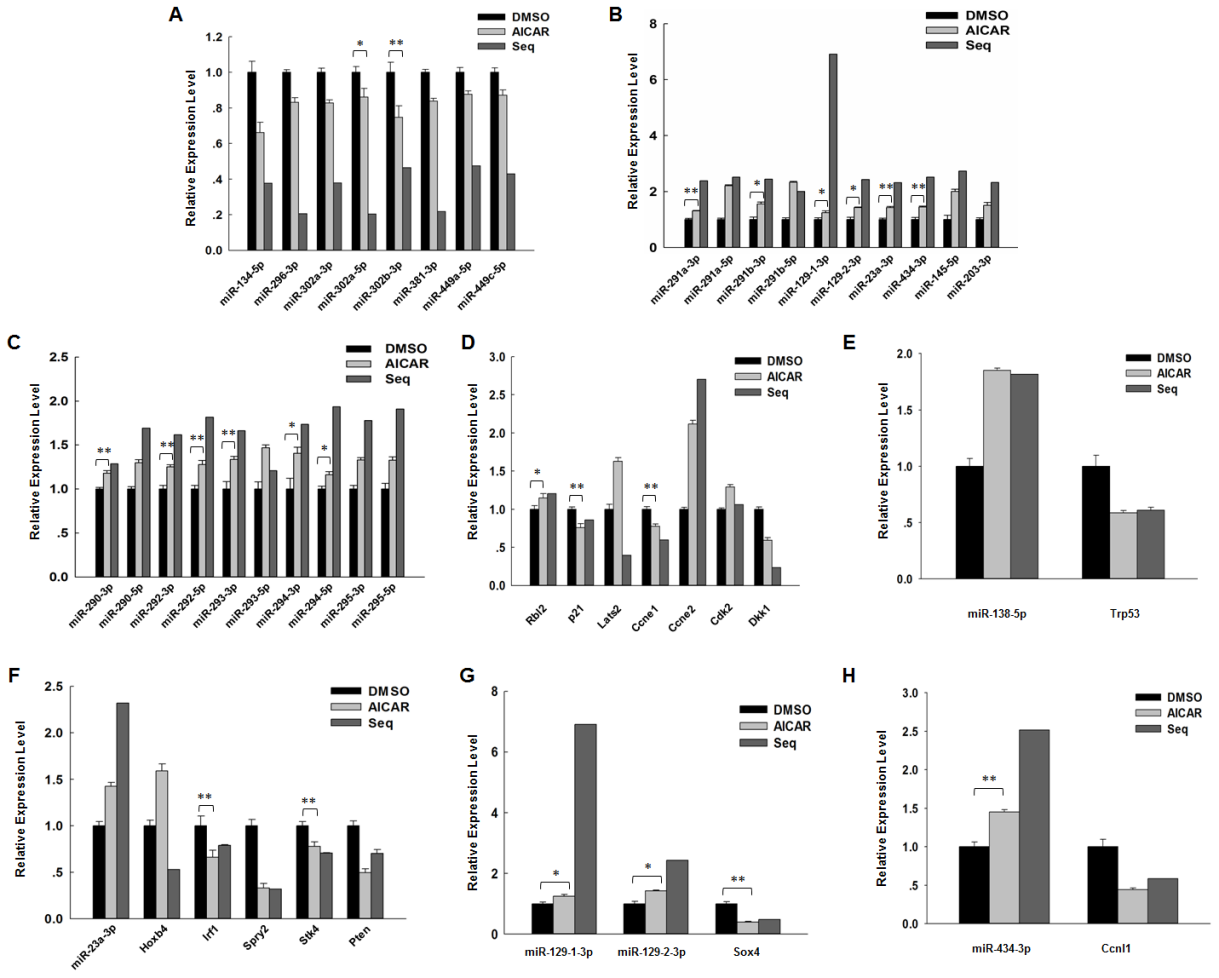
AICAR modulates the expression of multiple miRNAs associated with ES cell pluripotency. (A) Representative miRNAs that were down-regulated in the presence of 1 mM AICAR; this finding was validated by real-time PCR. J1 ES cells were cultured in medium with serum and 1,000 U/mL LIF in the presence or absence of 1 mM AICAR for 24 h. Mature miRNAs were transcribed, and the relative expression level of miRNAs was determined by real-time PCR. Data are presented as the mean ± SD of three independent experiments. (B) Representative miRNAs that were up-regulated in the presence of 1 mM AICAR were validated by real-time PCR. (C) Real-time PCR validation of miR-290 cluster after AICAR treatment. (D) Real-time PCR detection of the targets of miR-290 cluster in the presence of 1 mM AICAR. (E) The expression of miR-138-5p and its target Trp53 was validated by real-time PCR. (F) The expression of miR-23a-3p and its targets was validated by real-time PCR. (G) The expression of miR-129-1 and miR-129-2 and their target Sox4 was validated by real-time PCR. (H) The expression of miR-434-3p and its target Ccnl1 was validated by real-time PCR. (*: p<0.05; **: p<0.01).

Those down-regulated miRNAs included miR-134-5p, miR-296-3p, miR-381-3p, miR-449a-5p, miR-449c-5p, and miR-302 clusters. Among these miRNAs, miR-134 expression was elevated after retinoic acid (RA)-induced differentiation. The overexpression of this gene can enhance ES cell differentiation toward ectodermal lineages. MiR-296 was up-regulated after RA treatment of mouse ES cell. This gene can target the amino acid coding sequence of the crucial pluripotent gene, Nanog, thereby reducing its expression [21]. MiR-381 reportedly represses the expression of inhibitor of differentiation 1 (Id1), which functions in BMP signaling pathway to block ES cell neural differentiation [22]. MiR-449a and miR-449c are expressed during somitogenesis and neurogenesis, and these genes regulate Notch ligand *Delta-like 1* (Dll1) expression [23]. Mir-302 clusters are abundantly expressed in human ES cells and functioned in ES cell pluripotency maintenance and self-renewal [24]. MiR-302 has been demonstrated to be inhibited by BMP4. However, BMP4 was up-regulated by AICAR addition; therefore, the reduced miR-302 cluster expression may have resulted from increased BMP4 expression. The expression of these miRNAs was validated by real-time PCR, and the change tendency was consistent with our sequencing data, although their level of change was not exactly similar (Figure 2A).

By contrast, some miRNAs that are significantly up-regulated were also observed, including miR-290 cluster members, miR-291a and miR-291b, miR-129-1-3p, miR-129-2-3p, miR-23a-3p, miR-434-3p, miR-145-5p, and miR-203-3p. The mir-290 cluster was expressed abundantly in mouse ES cells; this cluster can promote mouse ES cell pluripotency and reprogramming by directly targeting key regulators of cell cycle and ensuring rapid G1-S transition [25]. Increased miR-129-1 and mir-129-2 expression was partly responsible for the reduced Sox4 expression, which was up-regulated during ES cell differentiation into neural stem cell [26]. The increased miR-23a expression may be caused by the down-regulation of its inhibitor C-myc [27]. Mir-434 is one of the highly expressed miRNA clusters in undifferentiated ES cells. Unfortunately, miR-203 and miR-145, which repress pluripotency-associated genes (i.e., Sox2 and Klf4), were also increased for unknown reasons [28]. Real-time PCR was performed to validate the expression of these miRNAs, and the result was consistent with the sequencing data (Figure 2B).

Considering the importance of the miR-290 cluster in mouse ES cell pluripotency maintenance, we also detected the expression level of other miR-290 cluster members by real-time PCR. Moreover, the result indicated that their expression levels were up-regulated after AICAR treatment; this finding suggested AICAR function in ES cell stemness maintenance (Figure 2C). Furthermore, we also examined the expression of miR-290-associated genes (Figure 2D). MiR-290 clusters were reported to be key regulators of the G1-S transition, and Cyclin E/Cdk2 has critical functions in this process [11]. Therefore, we detected the expression of Rbl2, Lats2, and p21, which are inhibitors of Cyclin E/Cdk2 pathway. We also detected the individual expression of Cyclin E and Cdk2. Our result shows that the expression of Rbl2, Lats2, and Cyclin E2 (Ccne2) was increased, whereas that of p21 and Cyclin E1 was decreased. Furthermore, Dkk1 was the miR-290 target, and this marker functioned by preventing ES cell differentiation into mesoderm and germ cell [25]. As expected, Dkk1 expression was decreased significantly, and this finding was consistent with our previous microarray data.

Aside from obtaining the aforementioned findings, we also performed mutual verification between miRNAs and their targets. The expression of miR-138 was increased, whereas its target Trp53 was down-regulated [29] (Figure 2E). MiR-23a was up-regulated after AICAR treatment, and the expression of all its targets, except Hoxb4, was reduced [27], [30]–[33] (Figure 2F). Similarly, the expression miR-129 and miR-434 was increased and their targets (i.e., Sox4 and Ccnl1, respectively) were down-regulated [26] (Figures 2G and 2H).

Overall, our study demonstrated that AICAR can modulate the expression of multiple miRNAs. Particularly, most differentiation -associated miRNAs were down-regulated, and pluripotency-associated miRNAs were up-regulated. These findings suggested AICAR function in ES cell pluripotency maintenance.

### Down-regulation of miR-134 is partly responsible for increased expression of pluripotency markers

**Figure 3:**
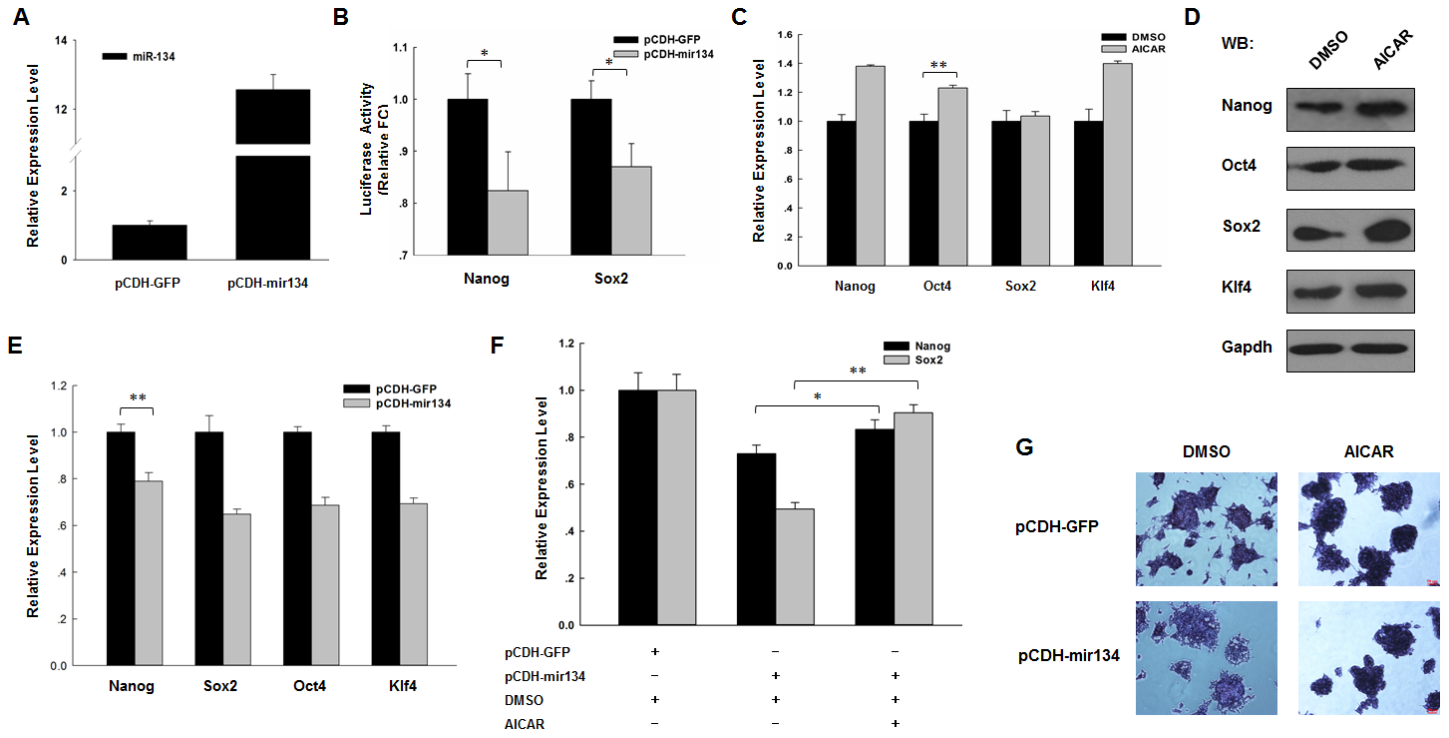
Down-regulation of miR-134 is partly responsible for increased expression of pluripotency markers. (A) MiR-134 expression vector pCDH-mir134 and its negative control were transfected into J1 ES cells, and the expression of miR-134 was detected by real-time PCR. (B) The luciferase reporters psi-Nanog or psi-Sox2 were co-transfected with miRNA expression vectors pCDH-mir134, and empty vector pCDH-GFP without insertion was used as control. 48 h after transfection, luciferase activity was detected using dual-luciferase reporter assay. (C) Real-time PCR validation of Nanog, Oct4, Sox2, and Klf4 in J1 ES cells in the presence or absence of 1 mM AICAR for 24 h using the comparative Ct method. Gapdh was used to normalize template levels. (D) Western blot analysis of pluripotency markers Nanog, Oct4, Sox2 and Klf4 in J1 ES cells in the presence of 1,000 U/ml LIF and with or without 1 mM AICAR for 24 h. Cell lysates were extracted and analyzed by western blot. Relative expression levels were comparing to Gapdh. (E) MiR-134 expression vector pCDH-mir134 and its negative control were transfected into J1 ES cells. 48 h after transfection, Nanog, Sox2, Oct4, and Klf4 expression was detected by real-time PCR. Gapdh was used to normalize template levels. (F) MiR-134 expression vector pCDH-mir134 and its negative control were transfected into J1 ES cells. 12 h after transfection, AICAR or DMSO was added to the cell culture medium, and Nanog and Sox2 expression was detected by real-time PCR. (G) MiR-134 expression vector pCDH-mir134 and its negative control were transfected into J1 ES cells. 12 h after transfection, AICAR or DMSO was added to the cell culture medium, and alkaline phosphatase staining of J1 cells were performed 24 hours later. (WB: western blot; *: p<0.05; **: p<0.01).

The expression level of miR-134 increased after RA- or N2B27-induced differentiation of mouse ES cells [34]. To investigate miR-134 function in AICAR-induced up-regulation of pluripotency markers, we constructed vector pCDH-mir134 that can express the miR-134 precursor to perform the succeeding experiment. Real-time PCR result showed J1 ES cells, which transfected with pCDH-mir134, exhibited high miR-134 expression level compared with those transfected with the control vector, pCDH-CMV-MCS-EF1-copGFP (pCDH-GFP) (Figure 3A). Nanog and Sox2 are direct targets of miR-134; thus, we constructed luciferase reporters that contain Nanog and Sox2 target sequence. Dual-luciferase reporter assay indicated that pCDH-mir134 can decrease the luciferase activity of reporters that contain the target sequence of miR-134 (Figure 3B).

Then, we detected the expression of major pluripotency markers Nanog, Oct4, Sox2, and Klf4 in the presence or absence of AICAR. Real-time PCR and western blot results indicated AICAR can increase the expression of those pluripotency markers (Figure 3C and 3D). After that, we overexpressed miR-134 in J1 ES cells and found that miR-134 overexpression can down-regulate the expression of four of the aforementioned pluripotency markers (Figure 3E). Furthermore, we added AICAR into the medium of J1 ES cells transfected with pCDH-mir134 to detect Nanog and Sox2 expression. The results indicated that AICAR can compromise miR-134 reduction on Nanog and Sox2 expression. In addition, AICAR cannot return to levels of cells transfected with control vector pCDH-GFP (Figure 3F).

To investigate the role of miR-134 in ES cells fate decision and highlight the function of AICAR, we transfected J1 ES cells with pCDH-mir134 and added AICAR to preform alkaline phosphatase activity assay. The results showed the expression of miR-134 can significantly decrease AP activity of ES cells and the addition of AICAR reversed this phenomena (Figure 3G). This functional experiment indicated the role of miR-134 in ES cells differentiation and the function of AICAR in their stemness maintenance.

In summary, these results indicated that AICAR can partly antagonize miR-134 function on ES cell differentiation.

### Decreased expression of Myc is modulatied by multiple miRNAs

Myc is one of the significantly down-regulated genes in J1 ES cells modulated by AICAR. Simultaneously, multiple miRNAs can be regulated by AICAR; thus, we attempted to explore the miRNAs that can target Myc and regulate its expression.

**Figure 4:**
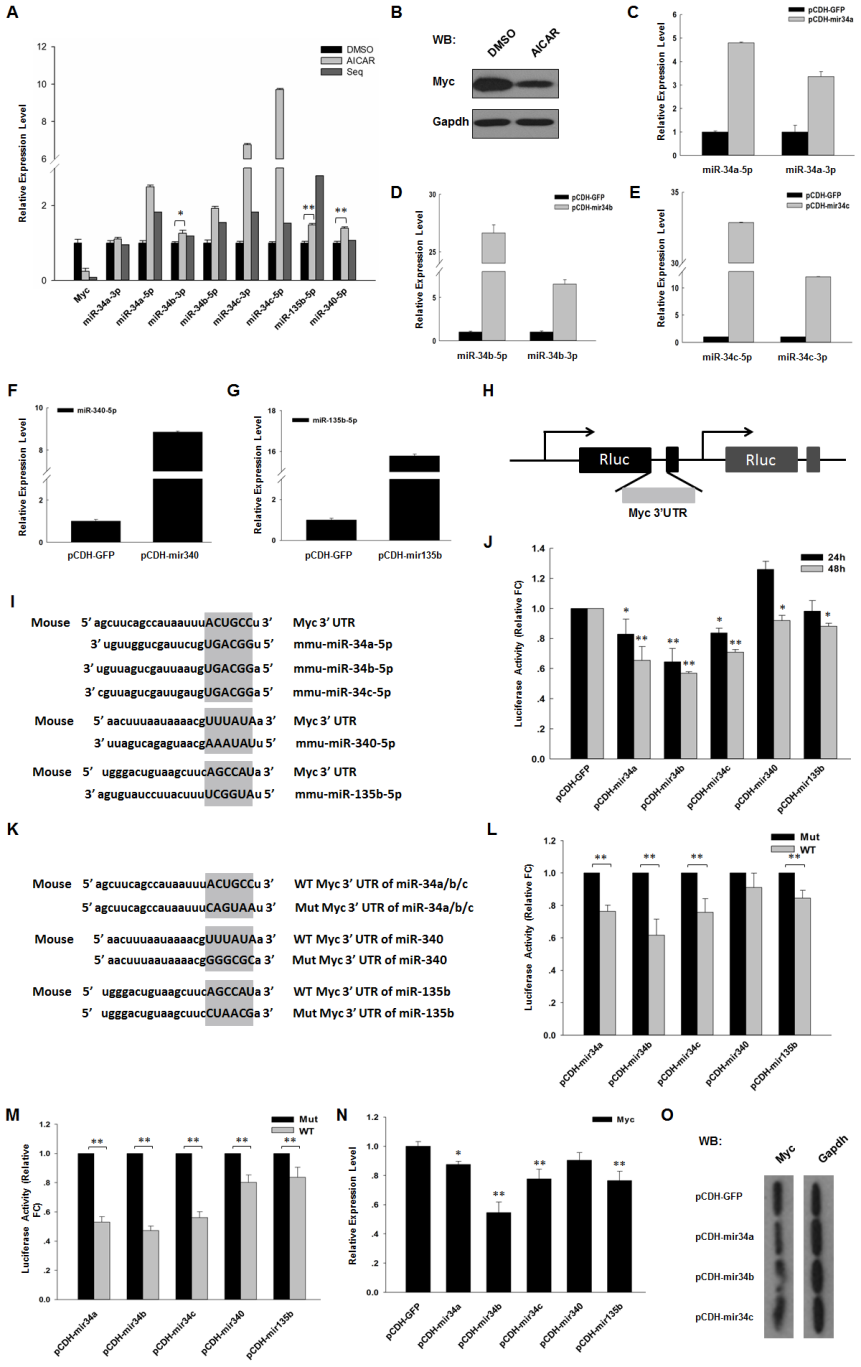
Several miRNAs targeted Myc and down-regulated its expression. (A) The expression of Myc and the predicted miRNAs was validated by real-time PCR in the presence of 1 mM AICAR. (B) Western blot analysis of Myc expression in J1 ES cells in the presence of 1,000 U/ml LIF and with or without 1 mM AICAR for 24 h. Cell lysates were extracted and analyzed by western blot. Relative expression level were comparing to Gapdh. (C/D/E/F/G) MiR-34a/34b/34c/340/135b expression vector pCDH-mir34a/34b/34c/340/135b and their negative control were transfected into J1 ES cells, and miR-34a/34b/34c/340/135b expression was detected by real-time PCR. (H) The schematic representation for construction of the firefly luciferase reporter vector. Myc 3′-UTR was inserted into psiCHECK-2 vector by XbaI and EcoRI sites. (I) Brief description of predicted miRNAs and their target sites on Myc 3′-UTR. (J) The luciferase reporters were co-transfected with miRNA expression vectors, and empty vector pCDH-GFP without insertion was used as control. 24 and 48 h after transfection, luciferase activity was detected using dual-luciferase reporter assay. (K) Brief description of mutated sites on Myc 3′-UTR. (L) The mutated luciferase reporters were co-transfected with miRNA expression vectors, and empty vector pCDH-GFP without insertion was used as control. 24 h after transfection, luciferase activity was detected using dual-luciferase reporter assay. (M) The mutated luciferase reporters were co-transfected with miRNA expression vectors, and empty vector pCDH-GFP without insertion was used as control. 48 h after transfection, luciferase activity was detected using dual-luciferase reporter assay. (N) The indicated miRNA expression vectors were transfected into J1 ES cells. 48 h after transfection, Myc expression was detected by real-time PCR. Gapdh was used to normalize template levels. (O) The indicated miRNA expression vectors were transfected into J1 ES cells. 48 h after transfection, cell lysates were extracted and analyzed by western blot. Relative expression level were comparing to Gapdh. (WB: western blot; *: p<0.05; **: p<0.01).

First, we predicted miRNAs that may target the 3′-UTR of Myc using TargetScan and miRanda database. By combining our sequencing data with the predicted result, we selected miR-34a, miR-34b, miR-34c, miR-340, and miR-135b to conduct further research. We performed real-time PCR to detect the expression level of Myc and of those predicted miRNAs. Real-time PCR results showed that Myc expression was significantly down-regulated, whereas the expression of miRNAs that may target Myc was increased (Figure 4A). The expression of Myc was also verified by western blot analysis (Figure 4B).

Subsequently, we constructed the miRNA expression vectors of those predicted miRNAs and named those as pCDH-mir34a, pCDH-mir34b, pCDH-mir34c, pCDH-mir340, and pCDH-mir135b. Real-time PCR results indicated that J1 ES cells, which transfected with those miRNA expression vectors, showed noticeably high miRNA expression level of corresponding miRNA. This finding was based on the comparison with cells transfected with the control vector pCDH-GFP (Figures 4C, 4D, 4E, 4F and 4G). Myc 3′-UTR sequence that contains putative miRNA binding sites of the aforementioned miRNAs were PCR amplified and cloned into MCS of dual-luciferase reporter vector psiCHECK-2 to engineer luciferase reporters psi-Myc-34, psi-Myc-340, and psi-Myc-135b (Figure 4H and 4I). In 293FT cells, the luciferase reporters were co-transfected with miRNA expression vectors, and empty vector pCDH-GFP without insertion was used as control. 24 h after transfection, only miR-34a, miR-34b, and miR-34c significantly reduced the luciferase activity of the Myc reporter between 20% and 35% compared to the control vector pCDH-GFP (Figure 4J). 48 h after transfection, miR-34a, miR-34b, and miR-34c can reach a 30% to 40% reduction, and miR-340 and miR-135b also showed slight reduction on the luciferase activity of Myc reporter (Figure 4J). By comparing the result obtained at 24 h with that at 48 h, we found that luciferase activity was much lower at 48 h than at 24 h. This finding indicated that miRNA regulation to its target was time dependent. Furthermore, we engineered mutated luciferase reporters with 6 base mutations in the predicted target sites of Myc 3′-UTR (Figure 4K). Compared with mutant reporters, miR-34a, miR-34b, miR-34c, and miR135b can repress the luciferase activity of Myc reporter by 10% to 40% at 24 h after transfection, and miR-340 showed no significant influence (Figure 4L). 48 h after transfection, miR-34a, miR-34b, and miR-34c showed approximately 50% reduction effect, whereas miR-340 and miR-135b showed approximately 20% reduction (Figure 4M). To study the repressed function of predicted miRNAs on Myc mRNA, we transfected these miRNAs into J1 ES cells and detected the endogenous expression level of Myc mRNA using real-time PCR 48 h after transfection. The result showed that miR-34a, miR-34b, and miR-34c can repress the expression by 15%, 55%, and 25%, respectively. Notably, miR-340 and miR-135b can also reduce Myc expression to the some extent (Figure 4N). Furthermore, we also detected the repression of miR-34a, miR-34b, and miR-34c on Myc protein expression. The result indicated all of this three miRNAs can reduce the expression of Myc and the function of miR-34b was most significantly (Figure 4O).

The aforementioned result suggested that miR-34a, miR-34b, and miR-34c not only target Myc in human derived cells as described previously [35]–[37], but also negatively regulate Myc expression in mouse species. In addition, Myc may also be the target of miR-340 and miR-135b, but this finding requires further investigation.

## Discussion

Because of their properties of pluripotency, self-renewal and unlimited proliferation, ES cells have great potentiality to be used in clinical medicine. However, the application of ES cells were limited partly because the mechanisms that ensure ES cell characteristics were not well elucidated, as well as the acquisition and culture of ES cell were rather difficult. So, discover the mechanisms in ES cells and ensure their properties during passage culture were urgent mission for us. A significant amount of transcriptional factors, such as Nanog, Oct4, Sox2, and Klf4, are composed of a center transcription network that has basic functions in ES cells. Signaling transduction pathways also have their functions in ES cell maintenance. Epigenetic modulation, including DNA methylation, histone modification, and non-coding RNA regulation, formed another regulatory system to maintain ES cell stemness [7], [38]. ES cells seemed to have their own unique epigenetic state, which can maintain their pluripotency and self-renewal, at the same time they can also immediately initiate differentiation during development. MiRNA is a type of non-coding RNA that has an essential function in ES cells through transcriptional and post-transcriptional regulation of gene expression.

In mouse ES cells, the miR-290 cluster is the most abundantly expressed miRNA, which measured up 60% to 70% of the total expressed miRNA [39]. In our study, almost all members of the miR-290 cluster were up-regulated after AICAR treatment. Cell cycle inhibitors p21 and Lats2 are direct targets of miR-290 cluster. As expected, p21 expression was decreased slightly. However, Lats2 was increased in our real-time detection, although this marker was decreased significantly in our sequencing data. Therefore, Lats2 was not only regulated by the miR-290 cluster, but also functioned in other cellular processes. Cyclin E/Cdk2 complex is direct modulator of G1/S transition and functions by phosphorylating its downstream proteins and promoting G1 progression. Our result showed that Cyclin E2 and Cdk2 expression was increased and Cyclin E1 was down-regulated. These findings may suggest different functions between Cyclin E1 and Cyclin E2. Rbl2 expression was not changed apparently. This finding indicated that AICAR may not influence de novo DNA methylation through the Rbl2 repression. Certainly, we do not rule out the possibility that AICAR can modulate DNA methylation through other methods. Dkk-1, which is directly targeted by the member of the miR-290 cluster, was down-regulated after ACAR treatment. This finding suggested the function of AICAR in repressing ES cell differentiation.

MiR-134, which was identified as a differentiation-associated miRNA, was decreasingly expressed after AICAR treatment. MiR-134 was first considered as a brain-specific miRNA that can regulate synapse development by targeting Limk1 [40]. Thereafter, miR-134 was found to be up-regulated after RA treatment, which caused Tay YM *et.al* to associate this RNA with ES cell differentiation. Further research indicated that miR-134 can modulate mouse ES cell differentiation by targeting Nanog and LRH1 and repressing their expression [34]. A recent study also found that miR-134 expression was correlated with tumor cell proliferation, survival, migration, and invasion [41]. Our data suggested that AICAR can down-regulated the expression of miR-134 in ES cells. Therefore, AICAR modulation on miR-134 expression may suggest the function of AICAR in ES cell pluripotency maintenance and cancer therapy.

Myc is a transcription factor that was enriched in ES cells and tumor cells. Yamanaka Laboratory first cultured iPS cells in 2006 using four specific factors, i.e., Oct4, Myc, Sox2, and Klf4 [42]. This experiment suggested the critical function of Myc in ES cells. However, in our study, Myc expression was seriously decreased in the presence of AICAR despite the up-regulation of other pluripotent factors, including Nanog, Sox2, Klf4, and Oct. In this study, we first identified that miR-34a, 34b, and 34c can repress Myc expression by directly targeting Myc 3′-UTR in mouse ES cells. In addition, miR-340 and miR-135 were also the potential miRNAs that can repress Myc expression. Thus, we propose that AICAR decreases Myc expression by modulating miRNA expression. The effect of AICAR on Myc expression may provide new insights into cancer therapy.

AICAR is a small molecule that can influence intracellular energy metabolism and stimulate cells in energy stress. Recently reported STAP cells proposed a more simpler and efficient pathway for somatic cell reprogramming. This research suggested external stimuli was conducive to the generation of pluripotent cells. In that way, we want to know whether the energy stress resulted from AICAR can facilitate reprogram efficiency? On the other hand, FSK, which was a cAMP agonist like AICAR, was used to produce iPS cells with other small molecules. So, if AICAR can replace FSK to accomplish the same task? In the study, our data demonstrated that AICAR can modulate miRNA expression, particularly miRNAs that participate in ES cell pluripotency maintenance. In addition, some important transcriptional factors, such as Sox2, Nanog and Myc, were also regulated by AICAR through its modulation on miRNA expression. All of these results suggested the function of AICAR on ES cell stemness maintenance and pluripotent cells generation. Thus, our study discovered a new mechanism of AICAR in ES cell pluripotency maintenance and provide insight for its usage in reprogramming and clinical medicine.

## Supporting Information

Table S1 **Real-time PCR primers.** List of all primers used for detection of microRNA or gene expression levels by real-time PCR.
(DOCX)

Table S2 **43 microRNAs identified to be significantly down-regulated after AICAR treatment.** Fold change (FC) values are provided in comparison with J1 ES cells treated by DMSO. (FC≤0.5, p≤0.01).
(DOCX)

Table S3 **59 microRNAs identified to be significantly up-regulated after AICAR treatment.** Fold change (FC) values are provided in comparison with J1 ES cells treated by DMSO. (FC≥2, p≤0.01).
(DOCX)
