# Supplementary material for: MicroRNA Modulation Induced by AICA Ribonucleotide in J1 Mouse ES Cells
Source: PLoS One. 2014 Jul 31;9(7):e103724. doi: 10.1371/journal.pone.0103724 (PMC4117590; doi:10.1371/journal.pone.0103724)
Supplement: Table S1 — Real-time PCR primers. List of all primers used for detection of microRNA or gene expression levels by real-time PCR. (DOCX) [file pone.0103724.s001.docx]

**Supplementary Table 1**. Real-time PCR primers. List of all primers used for detection of microRNA or gene expression levels by real-time PCR.

| **MicroRNA name** | | **Primer sequences** | |
| --- | --- | --- | --- |
| miR-145-5p | | GTCCAGTTTTCCCAGGAATCCCT | |
| miR-203-3p | | GTGAAATGTTTAGGACCACT | |
| miR-129-1-3p | | AAGCCCTTACCCCAAAAAGT | |
| miR-129-2-3p | | AAGCCCTTACCCCAAAAAGC | |
| miR-23a-3p | | ATCACATTGCCAGGGATTT | |
| miR-434-3p | | TTTGAACCATCACTCGA | |
| miR-135b-3p | | GTATGGCTTTTCATTCCTAT | |
| miR-138-5p | | GGAGCTGGTGTTGTGA | |
| miR-290-3p | | AAAGTGCCGCCTAGTTTTAAGC | |
| miR-290-5p | | ACTCAAACTATGGGGGCAC | |
| miR-291a-3p | | AAAGTGCTTCCACTTTGTGTGC | |
| miR-291a-5p | | CATCAAAGTGGAGGCCCTCTC | |
| miR-291b-3p | | AAAGTGCATCCATTTTGTTT | |
| miR-291b-5p | | GATCAAAGTGGAGGCCCTCTCC | |
| miR-292-3p | | GGGAAAGTGCCGCCAGG | |
| miR-292-5p | | ACTCAAACTGGGGGCTCTTTTG | |
| miR-293-3p | | AGTGCCGCAGAGTTTGTAG | |
| miR-293-5p | | ACTCAAACTGTGTGACATT | |
| miR-294-3p | | AAAGTGCTTCCCTTTTGTG | |
| miR-294-5p | | ACTCAAAATGGAGGCCCTA | |
| miR-295-3p | | AAAGTGCTACTACTTTTGAGTC | |
| miR-295-5p | | ACTCAAATGTGGGGCACACTTC | |
| miR-296-3p | | GAGGGTTGGGTGGAGGC | |
| miR-302a-3p | | TAAGTGCTTCCATGTTTTG | |
| miR-302a-5p | | GG ACTTAAACGTGGTTGT | |
| miR-302b-3p | | TAAGTGCTTCCATGTTTTAGTAG | |
| miR-134-5p | | TGTGACTGGTTGACCAG | |
| miR-381-3p | | TACAAGGGCAAGCTCTCTGT | |
| miR-449a-5p | | GGGTGGCAGTGTATTGT | |
| miR-449c-5p | | GGGAGGCAGTGCATT | |
| miR-340-5p | | GGGGTTATAAAGCAATGAG | |
| miR-34a-3p  miR-34b-5p  miR-34c-5p | | GGGGAATCAGCAAGTAT | |
| miR-34a-5p | | TGGCAGTGTCTTAGCTGG | |
| miR-34b-3p | | AATCACTAACTCCACTGCCATC | |
| miR-34b-5p | | GGGGAGGCAGTGTAA | |
| miR-34c-3p | | GGGGAATCACTAACCACACA | |
| miR-34c-5p | | GCAGTGTAGTTAGCTGATTGC | |
| **Gene name** | **Forward primer** | | **Reverse primer** |
| Gapdh | TGAACGGGAAGCTCACTGG | | TCCACCACCCTGTTGCTGTA |
| Sox4 | CGGCTGCATCGTTCTCTCC | | CGCTTCACTTTCTTGTCGGC |
| Hoxb4 | CGTGAGCACGGTAAACCCC | | GTGTTGGGCAACTTGTGGTC |
| Irf1 | ATGCCAATCACTCGAATGCG | | TTGTATCGGCCTGTGTGAATG |
| Spry2 | TCCAAGAGATGCCCTTACCCA | | GCAGACCGTGGAGTCTTTCA |
| Stk4 | TCATTCGGCTACGGAACAAGA | | GACCTGCGACTCCAAAGTCTG |
| Pten | TGGATTCGACTTAGACTTGACCT | | GCGGTGTCATAATGTCTCTCAG |
| Trp53 | GCTTTGAGGTTCGTGTTTGTG | | CTGGAGTGAGCCCTGCTGTC |
| Ccnl1 | GTGAACGTAACCAAACCCTGG | | CTAGCTGCAAGGTAGATACAAGC |
| Myc | AGCCCCTAGTGCTGCATGA | | TCCACAGACACCACATCAATTTC |
| Dkk1 | CGGGAACTACTGCAAAAATGGAAT | | CAAGGTTTTCAATGATGCTTTCCTC |
| Rbl2 | AACTTCCCCATGATTAGCGATG | | GGTTAGAACACTGAAGGGCATTT |
| Ccne1 | GTGGCTCCGACCTTTCAGTC | | CACAGTCTTGTCAATCTTGGCA |
| Ccne2 | ATGTCAAGACGCAGCCGTTTA | | GCTGATTCCTCCAGACAGTACA |
| Cdk2 | CCTGCTTATCAATGCAGAGGG | | TGCGGGTCACCATTTCAGC |
| Cdkn1a | CCTGGTGATGTCCGACCTG | | CCATGAGCGCATCGCAATC |
| Lats2 | GGACCCCAGGAATGAGCAG | | CCCTCGTAGTTTGCACCACC |
| Nanog | CACCCACCCATGCTAGTCTT | | ACCCTCAAACTCCTGGTCCT |
| Sox2 | GAGTGGAAACTTTTGTCCGAGA | | GAAGCGTGTACTTATCCTTCTTCAT |
