# Supplementary material for: MicroRNA Modulation Induced by AICA Ribonucleotide in J1 Mouse ES Cells
Source: PLoS One. 2014 Jul 31;9(7):e103724. doi: 10.1371/journal.pone.0103724 (PMC4117590; doi:10.1371/journal.pone.0103724)
Supplement: Table S2 — 43 microRNAs identified to be significantly down-regulated after AICAR treatment. Fold change (FC) values are provided in comparison with J1 ES cells treated by DMSO. (FC≤0.5, p≤0.01). (DOCX) [file pone.0103724.s002.docx]

**Supplementary Table 2**. 43 microRNAs identified to be significantly down-regulated after AICAR treatment. Fold change (FC) values are provided in comparison with J1 ES cells treated by DMSO. (FC ≤ 0.5, p ≤ 0.01)

| **miR-name** | **DMSO-std** | **AICAR-std** | **Fold Change** | **p-value** |
| --- | --- | --- | --- | --- |
| mmu-miR-1188-5p | 18.7596 | 8.155 | 0.434711 | 1.77E-20 |
| mmu-miR-1193-5p | 63.6448 | 25.7927 | 0.40526 | 3.71E-74 |
| mmu-miR-1197-3p | 5.5113 | 1.1379 | 0.206467 | 2.46E-15 |
| mmu-miR-130a-5p | 4.7694 | 1.8491 | 0.387701 | 2.48E-07 |
| mmu-miR-134-5p | 166.8226 | 62.9172 | 0.37715 | 5.92E-215 |
| mmu-miR-136-3p | 16.2689 | 7.3964 | 0.454634 | 1.42E-16 |
| mmu-miR-143-5p | 8.4789 | 3.556 | 0.419394 | 1.19E-10 |
| mmu-miR-149-3p | 13.2483 | 6.4008 | 0.483141 | 2.88E-12 |
| mmu-miR-154-3p | 48.0118 | 23.2798 | 0.484877 | 4.51E-40 |
| mmu-miR-181a-2-3p | 4.4514 | 0.7112 | 0.15977 | 8.24E-15 |
| mmu-miR-182-3p | 6.5712 | 2.3707 | 0.360771 | 1.48E-10 |
| mmu-miR-20b-3p | 1.9078 | 0.8534 | 0.447322 | 0.0042767 |
| mmu-miR-219-2-3p | 9.5388 | 4.7413 | 0.497054 | 9.92E-09 |
| mmu-miR-27a-3p | 194.3791 | 92.5977 | 0.476377 | 6.63E-163 |
| mmu-miR-296-3p | 139.3191 | 28.4953 | 0.204533 | 0 |
| mmu-miR-302a-3p | 1.3778 | 0.5215 | 0.378502 | 0.0050756 |
| mmu-miR-302a-5p | 9.0618 | 1.8491 | 0.204054 | 1.72E-24 |
| mmu-miR-3072-5p | 8.002 | 2.2758 | 0.284404 | 1.88E-16 |
| mmu-miR-323-3p | 1378.406 | 533.4446 | 0.387001 | 0 |
| mmu-miR-323-5p | 34.1276 | 14.3662 | 0.420955 | 4.73E-38 |
| mmu-miR-341-3p | 238.2044 | 59.1715 | 0.248406 | 0 |
| mmu-miR-344-3p | 2.5967 | 0.8534 | 0.328648 | 1.89E-05 |
| mmu-miR-3470a | 20.1374 | 10.0042 | 0.496797 | 7.45E-17 |
| mmu-miR-3473b | 48.1178 | 11.4266 | 0.237471 | 1.53E-108 |
| mmu-miR-370-3p | 7.1011 | 2.9396 | 0.413964 | 2.50E-09 |
| mmu-miR-377-5p | 14.5731 | 6.4008 | 0.43922 | 5.83E-16 |
| mmu-miR-379-3p | 4.4514 | 1.9439 | 0.436694 | 7.21E-06 |
| mmu-miR-379-5p | 211.6019 | 86.7659 | 0.410043 | 3.36E-237 |
| mmu-miR-381-3p | 1.7488 | 0.3793 | 0.216892 | 1.48E-05 |
| mmu-miR-382-3p | 317.7472 | 154.9459 | 0.487639 | 1.61E-251 |
| mmu-miR-410-5p | 8.7969 | 1.375 | 0.156305 | 3.07E-28 |
| mmu-miR-411-5p | 169.9492 | 84.253 | 0.495754 | 1.90E-130 |
| mmu-miR-431-3p | 27.3445 | 10.8102 | 0.395334 | 3.47E-34 |
| mmu-miR-449a-5p | 4.2924 | 2.0388 | 0.474979 | 5.29E-05 |
| mmu-miR-449c-5p | 5.2993 | 2.2758 | 0.429453 | 6.46E-07 |
| mmu-miR-466i-3p | 7.3661 | 2.987 | 0.405506 | 6.30E-10 |
| mmu-miR-540-3p | 35.6114 | 17.1161 | 0.480635 | 1.00E-30 |
| mmu-miR-540-5p | 12.7714 | 4.931 | 0.386097 | 2.09E-17 |
| mmu-miR-541-3p | 3.0206 | 0.9483 | 0.313944 | 1.99E-06 |
| mmu-miR-665-3p | 70.534 | 30.0599 | 0.426176 | 4.33E-75 |
| mmu-miR-758-5p | 16.2159 | 4.9784 | 0.307007 | 3.10E-29 |
| mmu-miR-770-3p | 19.8725 | 8.724 | 0.438999 | 3.16E-21 |
| mmu-miR-92a-2-5p | 280.758 | 114.6448 | 0.40834 | 2.62E-316 |
