# Supplementary material for: MicroRNA Modulation Induced by AICA Ribonucleotide in J1 Mouse ES Cells
Source: PLoS One. 2014 Jul 31;9(7):e103724. doi: 10.1371/journal.pone.0103724 (PMC4117590; doi:10.1371/journal.pone.0103724)
Supplement: Table S3 — 59 microRNAs identified to be significantly up-regulated after AICAR treatment. Fold change (FC) values are provided in comparison with J1 ES cells treated by DMSO. (FC≥2, p≤0.01). (DOCX) [file pone.0103724.s003.docx]

**Supplementary Table 3**. 59 microRNAs identified to be significantly up-regulated after AICAR treatment. Fold change (FC) values are provided in comparison with J1 ES cells treated by DMSO. (FC ≥ 2, p ≤ 0.01)

| **miR-name** | **DMSO-std** | **AICAR-std** | **Fold Change** | **p-value** |
| --- | --- | --- | --- | --- |
| mmu-miR-1196-5p | 0.7949 | 1.8017 | 2.266574 | 0.0055697 |
| mmu-miR-122-5p | 2.9146 | 25.4134 | 8.719344 | 4.38E-89 |
| mmu-miR-1247-5p | 0.5829 | 1.4224 | 2.440213 | 0.0086869 |
| mmu-miR-1249-3p | 0.5299 | 1.612 | 3.042083 | 0.0009648 |
| mmu-miR-1249-5p | 1.0069 | 3.4137 | 3.390307 | 2.24E-07 |
| mmu-miR-129-1-3p | 3.4976 | 24.1807 | 6.913512 | 7.25E-76 |
| mmu-miR-129-2-3p | 33.0148 | 80.1755 | 2.428471 | 5.09E-89 |
| mmu-miR-135b-5p | 2.5437 | 7.112 | 2.795927 | 3.18E-11 |
| mmu-miR-139-5p | 1.3248 | 2.8448 | 2.147343 | 0.0009103 |
| mmu-miR-145-5p | 6.2532 | 17.0687 | 2.729594 | 4.14E-24 |
| mmu-miR-149-5p | 0.5299 | 1.4224 | 2.68428 | 0.0045906 |
| mmu-miR-184-3p | 44.9912 | 94.115 | 2.091854 | 3.17E-78 |
| mmu-miR-1934-5p | 0.159 | 2.1336 | 13.41887 | 7.13E-10 |
| mmu-miR-1935 | 57.9216 | 151.8641 | 2.621891 | 3.21E-189 |
| mmu-miR-203-3p | 1.6958 | 3.9353 | 2.320616 | 2.39E-05 |
| mmu-miR-210-3p | 102.2239 | 279.5001 | 2.734195 | 0 |
| mmu-miR-210-5p | 8.002 | 16.9739 | 2.121207 | 7.48E-16 |
| mmu-miR-23a-3p | 256.4341 | 594.4652 | 2.318199 | 0 |
| mmu-miR-291a-3p | 481.4962 | 1147.16 | 2.382489 | 0 |
| mmu-miR-291a-5p | 1459.592 | 3666.691 | 2.512134 | 0 |
| mmu-miR-291b-3p | 52.9402 | 129.1532 | 2.439605 | 2.31E-143 |
| mmu-miR-291b-5p | 238.5754 | 478.9195 | 2.007414 | 0 |
| mmu-miR-3086-5p | 1.1659 | 4.1724 | 3.578695 | 3.50E-09 |
| mmu-miR-3107-5p | 1.4838 | 4.8835 | 3.291212 | 1.03E-09 |
| mmu-miR-324-5p | 8.5319 | 18.7756 | 2.200635 | 1.22E-18 |
| mmu-miR-328-3p | 0.4769 | 2.4655 | 5.169847 | 1.13E-07 |
| mmu-miR-345-5p | 5.9882 | 18.1118 | 3.024582 | 3.79E-29 |
| mmu-miR-365-3p | 12.1884 | 28.116 | 2.306784 | 1.34E-29 |
| mmu-miR-434-3p | 189.5037 | 476.8334 | 2.516222 | 0 |
| mmu-miR-455-3p | 0.159 | 1.5172 | 9.542138 | 1.15E-06 |
| mmu-miR-465a-3p | 6.2002 | 12.8964 | 2.079997 | 6.21E-12 |
| mmu-miR-465a-5p | 1.0599 | 2.3707 | 2.23672 | 0.0016298 |
| mmu-miR-465b-3p | 6.2002 | 12.8964 | 2.079997 | 6.21E-12 |
| mmu-miR-465c-3p | 6.2002 | 12.9912 | 2.095287 | 3.50E-12 |
| mmu-miR-466d-5p | 2.0137 | 5.0258 | 2.495804 | 3.40E-07 |
| mmu-miR-466n-5p | 2.0137 | 5.0258 | 2.495804 | 3.40E-07 |
| mmu-miR-467a-5p | 26.2316 | 56.9905 | 2.17259 | 5.72E-52 |
| mmu-miR-467b-5p | 26.2316 | 56.9905 | 2.17259 | 5.72E-52 |
| mmu-miR-484 | 7.2601 | 26.7884 | 3.689811 | 7.41E-53 |
| mmu-miR-486-3p | 0.212 | 2.0388 | 9.616981 | 1.32E-08 |
| mmu-miR-486-5p | 1.4308 | 4.8361 | 3.379997 | 6.91E-10 |
| mmu-miR-499-5p | 22.1512 | 89.184 | 4.026148 | 5.06E-187 |
| mmu-miR-500-3p | 1.4838 | 3.8879 | 2.620232 | 3.18E-06 |
| mmu-miR-5114 | 0.159 | 1.6595 | 10.43711 | 2.15E-07 |
| mmu-miR-574-3p | 0.371 | 1.4698 | 3.961725 | 0.0002736 |
| mmu-miR-574-5p | 1.3248 | 2.987 | 2.25468 | 0.0003531 |
| mmu-miR-669a-5p | 4.5574 | 10.6679 | 2.340786 | 1.87E-12 |
| mmu-miR-669d-5p | 1.3778 | 3.0344 | 2.202352 | 0.0004339 |
| mmu-miR-669o-5p | 1.6428 | 3.4612 | 2.106891 | 0.0003292 |
| mmu-miR-669p-5p | 4.5574 | 10.6679 | 2.340786 | 1.87E-12 |
| mmu-miR-671-5p | 0.212 | 1.4224 | 6.709434 | 1.50E-05 |
| mmu-miR-700-5p | 2.8616 | 5.8318 | 2.037951 | 6.61E-06 |
| mmu-miR-743b-3p | 0.9539 | 2.181 | 2.286403 | 0.002043 |
| mmu-miR-7b-5p | 0.265 | 1.0431 | 3.936226 | 0.0024438 |
| mmu-miR-871-3p | 0.265 | 1.0431 | 3.936226 | 0.0024438 |
| mmu-miR-874-3p | 0.7949 | 2.3707 | 2.982388 | 7.06E-05 |
| mmu-miR-880-3p | 1.1129 | 2.8448 | 2.556205 | 9.75E-05 |
| mmu-miR-881-3p | 1.7488 | 3.9353 | 2.250286 | 4.09E-05 |
| mmu-miR-883b-5p | 0.4239 | 1.8491 | 4.362114 | 1.87E-05 |
